# An Unreported Variant of the Rhomboideus Muscle With an Additional Cervical Origin in a Cross‐Breed Dog Cadaver

**DOI:** 10.1002/vms3.70693

**Published:** 2025-11-07

**Authors:** Younes Kamali

**Affiliations:** ^1^ Department of Basic Sciences Faculty of Veterinary Medicine Ferdowsi University of Mashhad Mashhad Iran

**Keywords:** anatomical variation, *Canis lupus familiaris*, cervical musculature, rhomboideus muscle, veterinary anatomy

## Abstract

The m. rhomboideus of domestic dogs typically originates from the dorsal median raphe of the neck and cranial thorax, with a distinct slip from the occiput, and is composed of the three parts: cervicis, thoracis and capitis. Here, an additional muscular slip was identified on the left side of an adult male cross‐breed dog cadaver. This ribbon‐shaped slip arose indirectly via tendinous fibres from the transverse process of the second cervical vertebra and extended caudodorsally to join the capital part near its scapular insertion. The slip was innervated by ventral branches of the cervical spinal nerves, consistent with the usual supply of the rhomboideus complex and distinct from the neighbouring m. serratus ventralis cervicis, despite their segmental overlap. Such a variant of the m. rhomboideus has not been previously reported in dogs. Recognition of incidental muscular variations, particularly with regard to their innervation patterns, provides insights into embryologic development and phylogenetic relationships in carnivores.

## Graphical abstract

A distinct muscular slip (arrows) was observed in the cervical region of a dog. It originated from the transverse process of the axis and joined the rhomboideus capitis (RCa) near its scapular insertion. The slip was innervated by a branch of the cervical ventral rami. This variant provides insights into anatomical variation and comparative morphology.

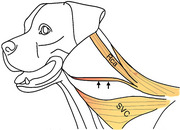

## 1 Introduction

In carnivores, the m. rhomboideus is divided into three parts: cervicis, thoracis and capitis. The m. rhomboideus capitis is a distinct muscle slip arising from the occipital bone and joining to the cervical portion caudally at the level of the fourth cervical vertebra. The cervical and thoracic parts are continuous, extending along the dorsal median raphe of the neck and cranial thorax, and inserting on the medial aspect of the dorsal scapular border and adjacent cartilage (Getty and Sisson 1975; Hermanson and de Lahunta 2018; Nickel et al. 1986; Singh 2018). At the insertion site, the rhomboideus lies in close contact with the m. serratus ventralis, which has cervical and thoracic parts originating from the transverse processes of the last five cervical vertebrae and the first seven or eight ribs, inserting on the facies serrata of the scapula (Hermanson and de Lahunta 2018).

Both the rhomboideus and serratus ventralis are supplied by ventral branches of the cervical spinal nerves, but their functional roles differ: the rhomboideus primarily stabilizes and elevates the scapula, whereas the serratus ventralis acts as a suspensory sling for the trunk (Hermanson and de Lahunta 2018). This overlap in innervation but divergence in function makes variations in these muscles particularly relevant for understanding neuromuscular organisation in the neck region.

Several anatomic variations of particular muscles in the neck region of domestic dogs and other carnivores have been described (Alić et al. 2014; de Souza Junior et al. 2018; Evans 1959; Giner et al. 2009; Terrado et al. 2005; Windle and Parsons 1897). However, no report has documented an accessory slip of the m. rhomboideus in the dog with an origin related to the transverse process of the axis. The present case describes such a variant, with emphasis on its innervation pattern and relationship to the neighbouring m. serratus ventralis cervicis.

## 2 Case Report

**FIGURE 1:**
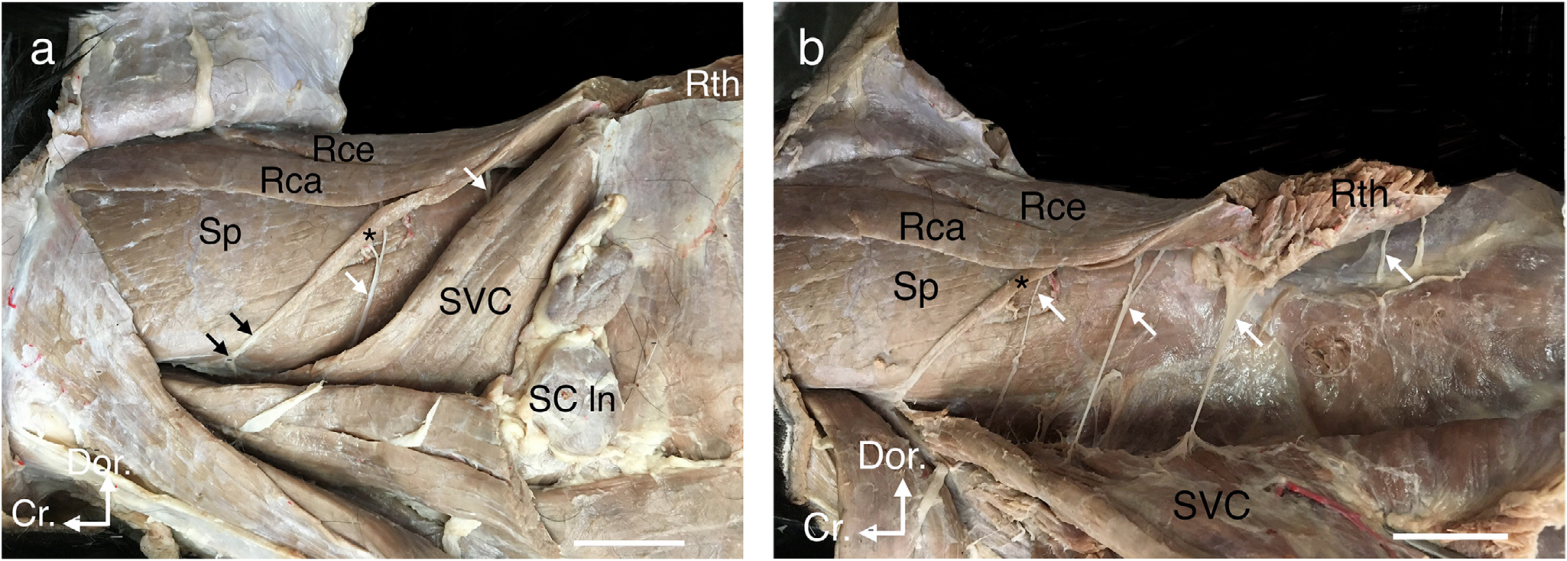
(a) Dissection of the left lateral side of the neck showing an additional muscle slip (*) of the m. rhomboideus, taking its origin via tendinous fibres (double arrowheads) from the transverse process of the second cervical vertebra to be attached to the capital part (RCa) near the scapula. (b) Dissection of the same side of the neck after reflecting the m. serratus ventralis cervicis (SVC) ventrally to show the ventral branches of the cervical nerves (white arrowheads), innervating several parts of the m. rhomboideus, including the additional muscle slip. RCe: m. rhomboideus cervicis; RTh: m. rhomboideus thoracis; SC ln: superficial cervical lymph nodes; Sp: m. splenius. Scale bar: 3 cm.

While dissecting the neck muscles of both sides of an adult male cross‐breed dog for teaching veterinary anatomy, a small, ribbon‐shaped additional muscle slip of the m. rhomboideus was detected on the left side only. The dog was an adult male, approximately 4 years old and weighing 18 kg. The dog was obtained from a private animal shelter for teaching purposes. It had been euthanised by intravenous injection of sodium pentobarbital (85 mg/kg) according to the recommendations of the AVMA Guidelines for the Euthanasia of Animals (2020 Edition) and embalmed via the left common carotid artery with a standard fixative solution containing 10% formaldehyde. The additional muscle slip arose from the ventral margin of the m. rhomboideus capitis caudally near the cranial angle of the scapula and extended craniaoventrally, lying at a distance between the rhomboideus and the m. serratus ventralis cervicis. It became tendinous and blended into the deep cervical fascia adjacent to the transverse process of the second cervical vertebra (Figure 1a). Qualitative assessment indicates the slip was approximately one‐third the width of the rhomboideus capitis at its midpoint. The slip was separated from the m. serratus ventralis cervicis by a distinct fascial plane, with no fibre interdigitation, and received innervation from ventral branches of the cervical spinal nerves, consistent with the rhomboideus complex (Figure 1b). No corresponding variation was observed on the right side.

## 3 Discussion

A handful of studies have addressed anatomic variations of selected neck muscles in domestic animals, so the introduction of novel variations remains of interest (Kamali 2022). In the present case, a ribbon‐shaped muscular slip extended from the m. rhomboideus capitis toward the transverse process of the axis, a pattern distinct from previously described variants in dogs or other carnivorans. (Alić et al. 2014) reported additional slips of the rhomboideus capitis located between the capital and cervical parts in two dogs, but these were positioned differently than in the current specimen (Alić et al. 2014). Among other canids, de Souza Junior et al. (2018) in Pampas foxes (*Lycalopex gymnocercus)* described underdevelopment or absence of the rhomboideus capitis, occasionally compensated by a thin slip arising from the m. serratus ventralis cervicis to the occiput (de Souza Junior et al. 2018). Unlike those cases, the present slip was strictly continuous with the rhomboideus capitis and separated from the serratus ventralis by a distinct fascial interval, without fibre sharing.

Innervation patterns suggest a closer relationship of the slip with the rhomboideus. Like the rhomboideus complex, it received ventral branches of the cervical nerves. Although the cervical portion of the serratus ventralis is also innervated by cervical branches (Getty and Sisson 1975; Nickel et al. 1986; Singh 2018), the clear separation of fibres and its continuity with the rhomboideus capitis favour its interpretation as a rhomboideus variant. For clarity of cervical innervation, the right side of the neck without the variant is shown (Figure S1), demonstrating the typical branching to the rhomboideus and serratus ventralis cervicis. Although both the rhomboideus and the serratus ventralis are classified as extrinsic muscles of the forelimb because of their scapular attachments, their embryological origins differ. The rhomboideus derives from cervical hypaxial myotomes, whereas the serratus ventralis originates from thoracic myotomes, with its cervical part representing a cranial extension (Saberi et al. 2017). These combined innervation and developmental features strengthen the conclusion that the present slip belongs to the rhomboideus.

From a phylogenetic standpoint, a comparable subdivision known as the m. rhomboideus profundus occurs in mustelids and procyonids, attaching ventrally to the transverse process of the atlas (Ercoli et al. 2015; Windle and Parsons 1897). The origin of the slip in the present case, via tendinous fibres from the axis, may represent a homologous remnant of this primitive condition retained from a predatory ancestor. Thus, incidental observations such as this are not only valuable for clinical and comparative anatomy but also contribute to a better understanding of the evolutionary and developmental patterns underlying variation in the cervical musculature of carnivorans.

## Author Contributions

Y.K. performed the experiments, analysed the data and wrote the manuscript.

## Funding

The author has nothing to report.

## Conflicts of Interest

The author declares no conflicts of interest.

## Supporting information




**Supplementary Figure S1**: Dissection of the right side of the neck showing the cervical innervation of the m. rhomboideus (R) and the m. serratus ventralis cervicis (SVC). Both muscles are innervated by ventral branches of the cervical spinal nerves (from C4 to C6, white arrowheads). This side did not present the additional muscular slip, and the image is provided to illustrate the typical pattern of cervical innervation for comparison with the variant observed on the left side. Note the most caudal cervical slip of the CVC, which is innervated by a branch (white curved arrow) of the n. thoracicus longus (TL). T1: thoracic spinal nerve 1; RCe: m. rhomboideus cervicis; RCa: m. rhomboideus capitis; SVT: m. serratus ventralis thoracis. Scale bar: 3 cm.

## Data Availability

The data presented in this study are available upon request from the corresponding author.
