# Supplementary figures and images for: An Unreported Variant of the Rhomboideus Muscle With an Additional Cervical Origin in a Cross‐Breed Dog Cadaver
Source: Vet Med Sci. 2025 Nov 7;11(6):e70693. doi: 10.1002/vms3.70693 (PMC12594214; doi:10.1002/vms3.70693)

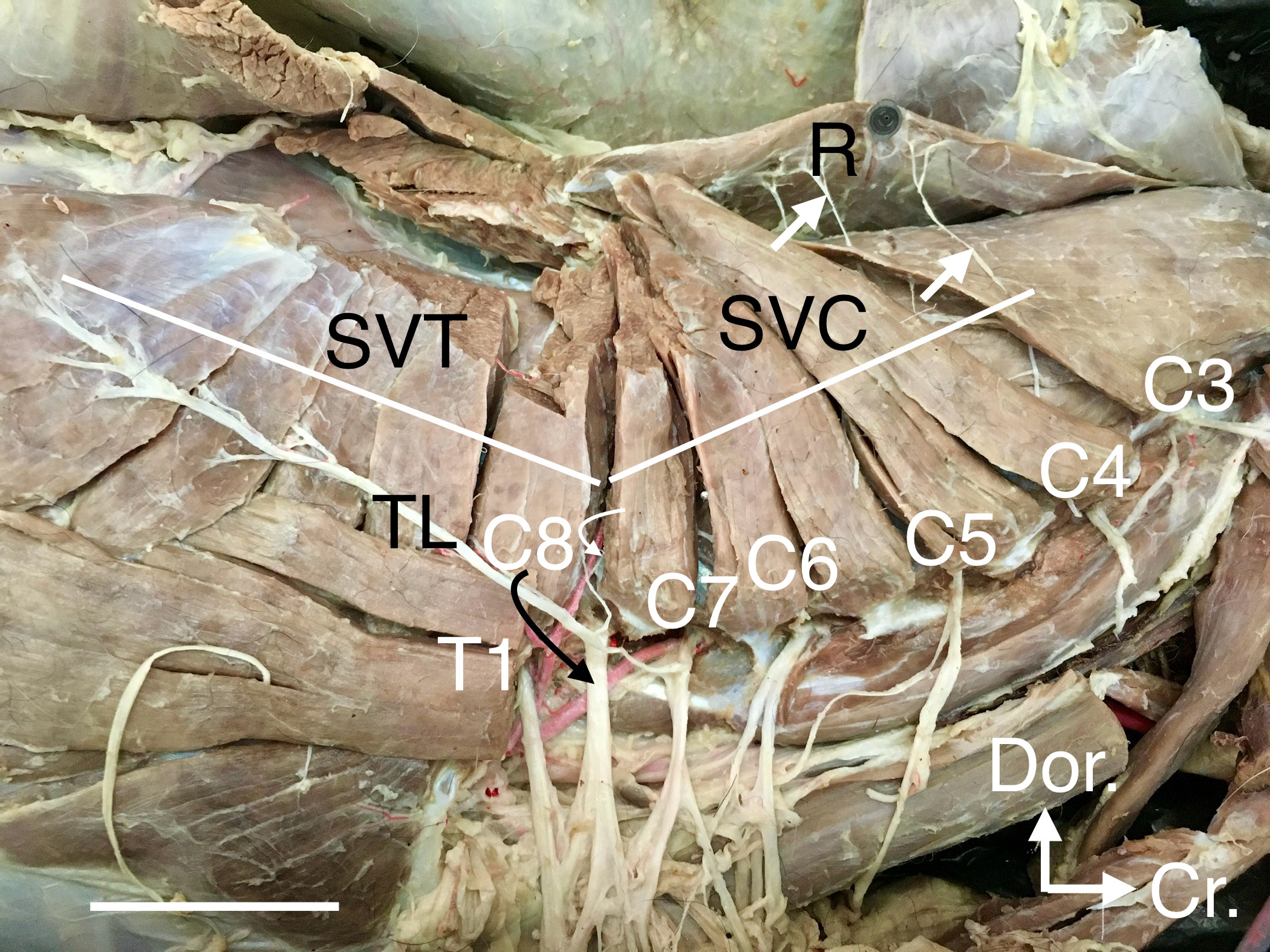

Supplement: Supplementary file 1 — Supplementary Figure S1: Dissection of the right side of the neck showing the cervical innervation of the m. rhomboideus (R) and the m. serratus ventralis cervicis (SVC). Both muscles are innervated by ventral branches of the cervical spinal nerves (from C4 to C6, white arrowheads). This side did not present the additional muscular slip, and the image is provided to illustrate the typical pattern of cervical innervation for comparison with the variant observed on the left side. Note the most caudal cervical slip of the CVC, which is innervated by a branch (white curved arrow) of the n. thoracicus longus (TL). T1: thoracic spinal nerve 1; RCe: m. rhomboideus cervicis; RCa: m. rhomboideus capitis; SVT: m. serratus ventralis thoracis. Scale bar: 3 cm. [file VMS3-11-e70693-s001.jpg]
